# Evaluation of conformity of preformed orthodontic archwires and dental arch form

**DOI:** 10.1590/2177-6709.24.1.044-052.oar

**Published:** 2019

**Authors:** Maheen Ahmed, Attiya Shaikh, Mubassar Fida

**Affiliations:** 1 Bakhtawer Amin Medical and Dental College (Multan, Pakistan).; 2 Liaquat College of Dentistry (Karachi, Pakistan).; 3 The AgaKhan University Hospital, Department of Surgery, Section of Dentistry (Karachi, Pakistan).

**Keywords:** Orthodontic wire, Relapse, Stability

## Abstract

**Introduction:**

The alterations in the arch form during treatment are dictated by the dimensions of the archwires.

**Objective:**

This study aimed to determine the mean arch dimensions of a sample of Pakistani subjects and to evaluate the conformity of preformed archwires with mandibular arch form.

**Methods:**

The dental records of 1,500 adult subjects were evaluated. The mandibular casts of 42 subjects (males = females = 21) with balanced facial profile, Class I occlusion, ideal overjet and overbite were included. Brackets were bonded on all teeth. Arch dimensions were evaluated at canines, first premolars, second premolars, first and second molars, with digital vernier calipers. The arch widths at the level of aforementioned teeth were evaluated on the digitized archwires, using the mean arch depths of the subjects.

**Results:**

In males, the archwires were found to be wider at canines and premolars, and significantly narrower at first (*p*< 0.001, 95% CI = 2.03 - 5.74) and second molars (*p*< 0.001, 95% CI = 2.29 - 7.73) as compared to the arch dimensions of the mandibular casts. In females, the archwires were significantly narrower at canines (*p*< 0.001, 95% CI = 1.4 - 3.97), and first (*p*= 0.02, 95% CI = 0.402 - 4.41) and second molars (*p*< 0.001, 95% CI = 1.76 - 6.13).

**Conclusion:**

No single commercially available archwires evaluated in the present study conformed to the arch dimensions of our subjects. Utilization of the currently available archwires may result in unwarranted modification of arch form, which may lead to unstable post-treatment teeth position.

## RESUMO

**Introdução:**

as alterações no formato da arcada dentária durante o tratamento são ditadas pelas dimensões do arco ortodôntico.

**Objetivo:**

o presente estudo teve como objetivos determinar a dimensão média das arcadas dentárias de pacientes paquistaneses e avaliar a conformidade entre diferentes arcos ortodônticos pré-fabricados e o formato da arcada inferior.

**Métodos:**

os registros odontológicos de 1.500 pacientes adultos foram analisados e, desses, foram selecionados modelos de gesso de 42 pacientes (21 de cada sexo, masculino e feminino) com perfil facial equilibrado, Classe I de Angle, sobressaliência e sobremordida adequadas. Foram, então, colados braquetes ortodônticos em todos os dentes. Utilizando um paquímetro digital, as dimensões das arcadas foram avaliadas nos seguintes dentes: caninos, primeiros pré-molares, segundos pré-molares, primeiros e segundos molares. Utilizando-se a profundidade média das arcadas dos pacientes, as larguras das arcadas, no nível dos dentes acima mencionados, foram comparadas aos arcos ortodônticos digitalizados.

**Resultados:**

nos homens, a largura do arco ortodôntico, em comparação às dimensões dos modelos de gesso inferiores, foi maior nos caninos e pré-molares e significativamente menor nos primeiros molares (*p*< 0,001, IC 95% = 2,03 - 5,74) e segundos molares (*p*< 0,001, IC 95% = 2,29 - 7,73). Nas mulheres, os arcos ortodônticos foram significativamente mais estreitos nos caninos (*p*< 0,001, IC 95% = 1,4 - 3,97), primeiros molares (*p*= 0,02, IC 95% = 0,402 - 4,41) e segundos molares (*p*< 0,001, IC 95% = 1,76 - 6,13).

**Conclusão:**

nenhum dos arcos ortodônticos comercialmente disponíveis avaliados no presente estudo esteve em conformidade com as dimensões das arcadas dos pacientes da amostra. O uso dos arcos ortodônticos atualmente disponíveis pode resultar em modificações indesejáveis no formato da arcada, e pode levar à instabilidade na posição dos dentes após o tratamento.

## INTRODUCTION

Relapse of the corrected malocclusion, being one of the biggest dilemmas of orthodontic treatment, has consistently been a topic of discussion in the orthodontic literature. A review of the literature showed that only 30% of the treated cases retained their alignment ten years post retention, which is further reduced to only 20% at the time of the twenty years follow up.^1^ Freitas et al^2^ reported a mean mandibular crowding of 1.96 mm (26.54%) over long term during the post-retention phase. Factors that may affect relapse include the continued growth of jaws, severity of original malocclusion, incisor position, arch form and mode of retention.^3^ Amongst these, the modification of original arch form during orthodontic treatment is considered to be one of the most common causes of relapse.^4^ ^,^ ^5^

The dental arch form is initially shaped by the configuration of the supporting bone. After the eruption of teeth, it is further modified by the surrounding musculature and functional forces.^6^ If this arch form is altered during orthodontic treatment, there is a tendency for it to return to its pretreatment shape. Various studies have reported the return of the canine and molar widths to pretreatment position during the post-retention phase if the original arch form is modified.^7^ ^-^ ^10^ Hence, the maintenance of original arch form rather than arch modification is generally recommended to reduce the relapse tendency.

The modern straight-wire appliance consists of brackets with built-in prescriptions and archwires. The archwires come in various sizes and shapes, according to different manufacturers’ specifications. These wires are designed according to the mean arch dimensions derived from a specific population. When a particular form of archwire is used, the existing arch form of an individual is altered to match the shape of that particular wire. Moreover, the arch form tends to differ among various ethnic and age groups.^11^ ^,^ ^12^ As the dental arch form may vary due to aforementioned reasons, there is no consensus on the ideal shape and size of the archwire. Further, the majority of the commercially available archwires are manufactured in USA, China and other countries according to their population standards.^13^ Therefore, it is critical to select the appropriate archwire form for each case. The present study aimed to determine the arch dimensions in males and females of a sample of Pakistani subjects. These mean arch dimensions were further used to identify the commercially preformed archwires currently available in Pakistan that best conform to the arch dimensions of these subjects. 

## MATERIAL AND METHODS

The study was conducted on the mandibular casts of 42 adult subjects (males = 21; females = 21) aged 18-30 years meeting the following inclusion criteria: well-balanced facial profile, Class I molar, canine and incisor relationship, and an ideal overjet and overbite. Subjects with any dental prosthesis, arch length discrepancy greater than 2 mm or history of facial/dental trauma were excluded. 

The sample size was calculated using the findings of Jonathan et al,^14^ who reported a mean canine width of 35.22 ± 1.54 mm in males and 33.49 ± 1.49 mm in females. Keeping α = 0.05 and power of the study as 90 %, a sample size of 17 subjects in each group was required. This number was inflated to 21 subjects in each group. This resulted in a total sample of 42 subjects. The dental records of 1,500 adult patients with complete dentition (excluding third molars) presenting to the dental clinics at a tertiary care hospital and university were evaluated to finally obtain the sample of 42 subjects meeting the aforementioned inclusion criteria.

### Determination of arch form dimensions of subjects without bracket-archwire assembly

A sharply trimmed lead pencil (Staedtler HB, Nürnberg, Germany) was used to mark the facial axis (FA) points on all the teeth, with the aid of a bracket positioning gauge (3M Unitek, Monrovia, Calif). The marked points were remeasured to avoid any discrepancy. A plastic transparent ruled grid was then placed on each mandibular cast. The purpose was to provide a stable base to place the measuring instrument, and grids served as guidelines to avoid measurement errors, especially when measuring the arch depths. A digital vernier caliper (Mitutoyo, Kawasaki, Japan) was used to perform the following linear measurements:

1. Arch width: Canine (IC), first premolar (IP1), second premolar (IP2), first molar (IM1) and second molar (IM2) widths, measured as the distance between the FA points on canines, first premolars, second premolars, first molars and second molars, respectively.

2. Arch depth: Canine, first premolars, second premolars, first molars and second molars widths, measured as the perpendicular distance from the midway point between the line connecting the FA points on the central incisors and the line connecting the FA points on the respective teeth.

### Determination of arch form dimensions of subjects with bracket-archwire assembly

**Figure 1:**
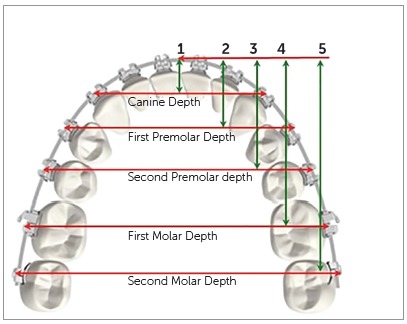
Variables measured on mandibular cast with bracket-archwire assembly.

The metal brackets (Roth 0.022 x 0.028-in slot; 3M Unitek, Monrovia, Calif) were then bonded on teeth of all the mandibular casts by the main investigator on the FA points. These brackets were tied with elastomeric ligatures to a 0.017 x 0.025-in stainless steel (SS) wire (3M Unitek, Monrovia, Calif). The aforementioned wire size was used as all the commercially available archwires evaluated in the current study were of the same dimension. The bracket axis (BA) point was determined as the middle of the archwire slot, in line with the FA point on each tooth. The following linear measurements were then made (Fig 1):

1. Arch width: Canine (CW), first premolar (PW1), second premolar (PW2), first molar (MW1), second molar (MW2) widths, measured as the distances between BA points on canines, first premolars, second premolars, first molars and second molars, respectively.
2. Arch depth: Canine (CD), first premolar (PD1), second premolar (PD2), first molar (MD1) and second molar (MD2) depths, measured as the perpendicular distance from the point midway on the archwire between the line connecting the BA points on the central incisors and the line connecting the BA points on the respective teeth.

### Determination of arch width of commercially available preformed archwires

**Figure 2:**
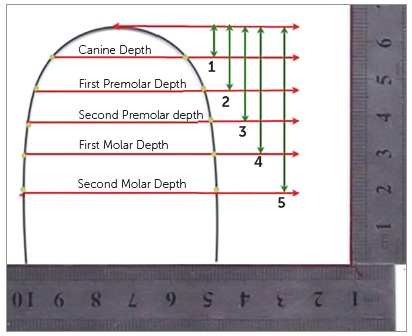
Variables measured on preformed archwires using mean arch depths as measured on mandibular cast.

A total of 15 different archwires from 6 different manufacturers of 5 shapes were analyzed (Table 1). The archwires included in the study were the ones that are commonly being used at various orthodontic centers in Pakistan. To reduce the risk of bias, all the archwires were given a specific code prior to the analysis. These archwires were scanned using a Canon flatbed scanner (CanoScan LiDE 210). In order to minimize the magnification error, a millimeter ruler was placed along with the archwires during scanning. The Adobe Photoshop software (version 7.0, Photoshop, Adobe, San Jose, Calif) was further used to deduce any magnification error. The mean canine, first premolar, second premolar, first molar and second molar depths as measured from the mandibular casts of 42 subjects were used as reference to measure the canine (CW), first premolar (PW1), second premolar (PW2), first molar (MW1) and second molar (MW2) widths on the archwires. The Adobe Photoshop software was used for the measurements on the digitized archwires (Fig 2). The readings were rounded off to two decimal points. All the measurements on the casts and archwires were repeated twice to rule out any measurement error.

**Table 1:** Shapes of arch wires and manufacturer.

	Bonewill-Hawley	Ovoid	Tapered	Square	Brader
Shape	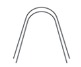	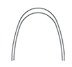	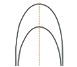	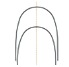	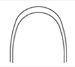
Brand	All Star	Ortho Organizer	Ortho Organizer	Ortho Organizer	Orthoclassic
	Orthocare	Orthoclassic	3M	Orthoclassic	All Star
		3M		3M	Dentsply
		All Star			Orthocare

### Statistical analysis

Ten dental casts and archwires were randomly selected and remeasured by the main investigator to determine the intraexaminer reliability (Table 2). The result showed a high correlation between the two sets of readings. The archwires were scanned and arch dimensions were evaluated using Adobe Photoshop software. Ten archwires were randomly selected and were manually measured by hand using a digital vernier calliper. The same archwires were then remeasured on Adobe Photoshop software, to determine the absolute error (Table 2). The Shapiro-Wilk test was used to check the normality of the data, and showed a non-normal distribution. The Mann-Whitney U test was used to compare the mean arch dimensions between genders. The same statistical analysis was used to compare the mean arch widths of the mandibular arch and archwires. 

**Table 2:** Intra-examiner reliability.

Parameter	First reading (n=10) Mean ± SD (mm)	Second reading (n=10) Mean ± SD (mm)	Mean difference ± SD	P-value**	ICC
Arch width measurements made on dental casts					
Canine Width	29.88 ± 2.20	29.95 ± 2.21	0.01 ± 0.33	0.926	0.987
First Premolar Width	41.19 ± 2.70	40.90 ± 2.70	0.09 ± 0.46	0.556	0.964
Second Premolar Width	42.94 ± 2.60	42.60 ± 2.50	0.02 ± 0.63	0.922	0.987
First Molar Width	49.67 ± 2.83	50.00 ± 2.94	0.17 ± 0.73	0.484	0.940
Second Molar Width	53.00 ± 2.69	53.50 ± 2.71	0.09 ± 0.78	0.737	0.989
Archwire measurements made on Adobe Photoshop					
Canine Width	27.87 ± 2.11	27.88 ± 2.01	0.70 ± 0.36	0.553	0.987
First Premolar Width	39.85 ± 1.52	39.95 ± 1.85	0.29 ± 0.97	0.371	0.937
Second Premolar Width	45.56 ± 3.99	45.58 ± 3.70	0.34 ± 0.84	0.232	0.946
First Molar Width	53.43 ± 2.10	53.60 ± 2.11	0.33 ± 0.60	0.115	0.979
Second Molar Width	59.38 ± 5.32	59.47 ± 5.27	0.50 ± 0.35	0.06	0.991
Archwire measurements made by hand and then repeated on Adobe Photoshop					
Canine Width	29.20 ± 1.94	27.87 ± 2.11	0.12 ± 0.51*	0.474	0.977
First Premolar Width	40.20 ± 2.44	39.85 ± 1.52	0.04 ± 0.74*	0.868	0.965
Second Premolar Width	42.33 ± 2.72	45.56 ± 3.99	0.61 ± 0.97*	0.077	0.935
First Molar Width	48.83 ± 2.98	53.43 ± 2.10	0.03 ± 0.87*	0.915	0.958
Second Molar Width	52.36 ± 2.61	59.38 ± 5.32	0.01 ± 0.64*	0.962	0.972

n = 10; SD = Standard Deviation; ICC = Intraclass Correlation Coefficient. *Mean absolute error. **Paired sample t-test.

## RESULTS

The mean arch dimensions as measured from the FA point without the bracket wire assembly on the mandibular casts are shown in Table 3. All the arch dimensions were found to be larger in males as compared to the females.

**Table 3:** Mean arch dimensions in males and females without bracket-archwire assembly.

Parameter	Males (n = 21) Mean ± SD (mm)	Females (n = 21) Mean ± SD (mm)
Intercanine Width (IC)	28.09 ± 2.16	28.23 ± 1.48
First Premolar Width (IP1)	37.64 ± 1.35	37.29 ± 1.73
Second Premolar Width (IP2)	43.8 ± 2.16	42.73 ± 2.25
First Molar Width (MW1)	50.73 ± 1.83	47.58 ± 5.75
Second Molar Width (MW2)	56.38 ± 4.13	54.93 ± 2.72
Canine Depth	6.46 ± 0.94	5.81 ± 0.75
First Premolar Depth	12.6 ± 2.04	11.96 ± 2.19
Second Premolar Depth	19.11 ± 2.22	18.98 ± 1.74
First Molar Depth	27.09 ± 2.12	26.01 ± 1.83
Second Molar Depth	38.47 ± 2.73	36.97 ± 2.88

n = 42; SD = Standard Deviation.

The arch dimensions as measured from BA point with the bracket-archwire assembly were compared between the two genders (Table 4). The CD (*p*= 0.035, 95% CI = 0.181 - 1.77) and MW1 (*p*< 0.033, 95% CI = 0.685 - 3.25) showed significant differences.

**Table 4:** Comparison of arch dimension parameters between males and females with bracket-archwire assembly.

Parameter	Males (n = 21) Mean ± SD (mm)	Females (n = 21) Mean ± SD (mm)	P-value	95 % Confidence Interval	
				Lower limit	Upper limit
Canine Width (CW)	28.54 ± 1.99	30.97 ± 1.85	0.206	-3.6	1.2
First Premolar Width (PW1)	40.24 ± 1.35	39.89 ± 1.73	0.614	-0.655	1.35
Second Premolar Width (PW2)	45.92 ± 3.73	45.78 ± 2.28	0.515	-1.81	2.11
First Molar Width (MW1)	54.04 ± 1.83	52.07 ± 2.18	0.033*	0.685	3.25
Second Molar Width (MW2)	58.67 ± 4.13	57.20 ± 2.72	0.394	-0.823	3.74
Canine Depth (CD)	5.97 ± 0.94	5.33 ± 0.74	0.035*	0.181	1.77
First Premolar Depth (PD1)	14.42 ± 2.04	13.77 ± 2.19	0.273	-0.726	2.02
Second Premolar Depth (PD2)	20.92 ± 2.33	20.79 ± 1.75	0.676	-1.13	1.4
First Molar Depth (MD1)	28.90 ± 2.12	27.82 ± 1.83	0.127	-0.172	2.34
Second Molar Depth (MD2)	40.28 ± 2.74	38.78 ± 2.89	0.053	-0.289	3.3

n = 42; SD = Standard Deviation. *p < 0.05, ** p < 0.01; Mann-Whitney U test.

**Figure 3:**
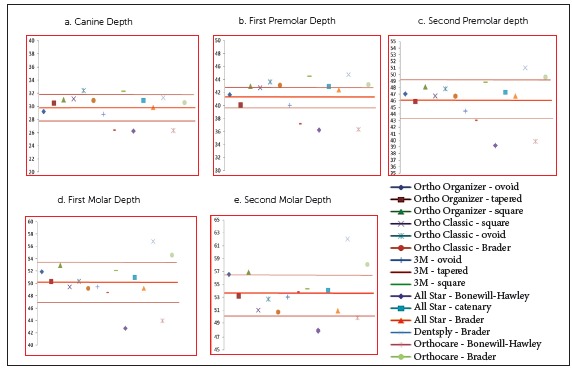
Males: comparison of individual preformed archwires with mean arch widths, including bracket-archwire assembly.

In males, the mean CW (*p*= 0.030, 95% CI = 0.027 - 0.034) and PW1 (*p*= 0.039, 95% CI = 0.033 - 0.040) as measured on the preformed archwires were found to be wider as compared to the mean arch widths of the subjects included in the study. In contrast, the mean MW1 (*p*< 0.001, 95% CI = 2.03 - 5.74) and MW2 (*p*< 0.001, 95% CI = 2.29 - 7.73) were found to be narrower (Table 5). The comparison of each individual preformed archwire with the mean arch width of the mandibular cast in male subjects is shown in Figure 3.

**Table 5:** Comparison of arch width dimensions between preformed archwires and mandibular arch including bracket-archwire assembly.

Gender	Parameter	Mandibular arch (Mean ± SD) (mm)	Preformed archwire (n = 15) (Mean ± SD) (mm)	P-value	95 % Confidence Interval	
					Lower limit	Upper limit
Male (n=21)	Canine Width (CW)	28.54 ± 1.99	29.84 ± 2.07	0.030*	0.027	0.034
	First Premolar Width (PW1)	40.24 ± 1.35	41.42 ± 2.84	0.039*	0.033	0.040
	Second Premolar Width (PW2)	45.92 ± 3.73	46.13 ± 3.31	0.874	-2.66	2.23
	First Molar Width (MW1)	54.04 ± 1.83	50.15 ± 3.58	0.001*	2.03	5.74
	Second Molar Width (MW2)	58.67 ± 4.13	53.66 ± 3.60	0.001*	2.29	7.73
Female (n=21)	Canine Width (CW)	30.97 ± 1.85	28.28 ± 1.86	< 0.001*	1.4	3.99
	First Premolar Width (PW1)	39.89 ± 1.73	40.98 ± 2.84	0.077	-2.7	0.51
	Second Premolar Width (PW2)	45.78 ± 2.28	46.05 ± 3.31	0.571	-2.2	1.65
	First Molar Width (MW1)	52.07 ± 2.18	49.66 ± 3.53	0.019*	0.402	4.41
	Second Molar Width (MW2)	57.20 ± 2.72	53.26 ± 3.55	0.001*	1.76	6.13

n = 42; SD = Standard Deviation. *p < 0.05; ** p < 0.01; Man-Whitney U test.

**Figure 4:**
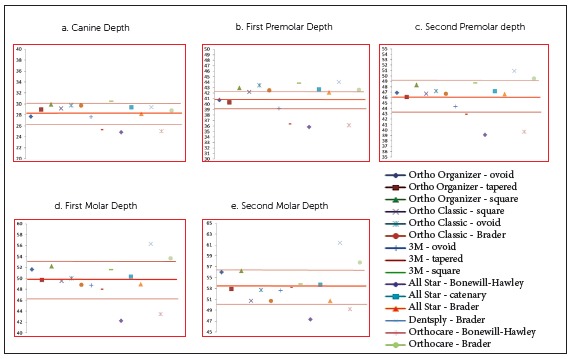
Females: comparison of individual preformed archwires with mean arch widths, including bracket-archwire assembly

In females, the mean CW (*p*< 0.001, 95% CI = 1.4 - 3.97), MW1 (*p*= 0.02, 95% CI = 0.402 - 4.41) and MW2 (*p*< 0.001, 95% CI = 1.76 - 6.13) of the preformed archwires was found to be narrower as compared to the mean arch widths of the subjects (Table 5). Different archwires were then compared individually with the mean arch dimensions of the mandibular arch in both males and females separately (Figs 3 and 4). In the current study, the Brader archwire shape in both males and females most closely conformed to the mean canine width of our subjects. The narrow shape of archwires, i.e. Bonewill-Hawley, ovoid and tapered forms, more closely conformed to the first premolar width in males and females, respectively. In the second premolar, first molar and second molar region, arch forms like square, ovoid and Brader more closely matched our subjects.

## DISCUSSION

The dimensions of an arch tend to vary between genders and among various ethnicities.^15^ ^-^ ^17^ In the present study, the arch width at canines, first premolars and first molars was found to be similar to that of Turkish subjects.^15^ In contrast, the Colombians and Koreans were found to have wider arch dimensions as compared to our subjects.^16^ ^,^ ^17^ Hence, it is impossible to define an ideal generalized arch form that may be used as a universal template for all populations.

To minimize the changes in arch form during orthodontic treatment, the form of a particular archwire may be modified according to an individual’s arch form. This is only possible if wires with good formability such as beta titanium (TMA) or stainless steel (SS) are used. The nickel-titanium (NiTi) wires possess only 10-28% of the stiffness of the SS wires. These wires are difficult to modify due to superelasticity and poor formability. Hence, if these preformed wires do not conform to an individual’s arch shape, they may result in undue modification of the original arch form. This has esthetic and stability implications.^18^ Secondly, the preformed archwires are manufactured on mean dimensions derived from specific populations. These may not conform to our population norms due to ethnic variations.^13^ A survey of the pertinent literature showed that no such study has been conducted on Pakistani population. Therefore, the purpose behind this study was to evaluate which of the preformed archwires conformed to arch forms in our sample of Pakistani subjects.

The mandibular arch has therapeutic limitations during orthodontic treatment due to surrounding musculature and occlusal forces.^19^ Secondly, maintaining pretreatment mandibular intercanine width is essential to minimize the orthodontic relapse tendency.^9^ For the aforesaid reasons, the mandibular arch form was analyzed in the current study. Moreover, various studies have reported changes in arch dimensions with age.^6^ Hence, in the present study only adult subjects were included.

In the current study, all the arch dimensions except canine width were found to be narrower in females as compared to the males. This may be due to the reason that males have morphologically larger jaw size as compared to females, due to gender dimorphism.^20^ The canine depth and first molar width showed significant difference between the two groups. As variations in arch depth may affect arch width of archwires, therefore all the preformed archwires were compared separately for males and females. 

The canine and molar widths with the bracket-archwire assembly when compared to studies conducted on other populations were found to be variable.^21^ ^,^ ^22^ The differences in results among various studies may be due to variations in thickness of bracket-wire assembly. Hence, the canine width reported in all the studies is not an accurate representation of that population’s arch width and differences may occur if a bracket of different thickness is used. To avoid this variation, the actual canine and molar widths were also reported in the current study excluding the bracket-archwire assembly. These findings may be used and modified in cases where a bracket system with different bracket base thickness is used. 

All the preformed archwires were found to be wider in the canine and premolars region as compared to the mean CW, PW1 and PW2 of the mandibular arch in males. On the contrary, when the molar widths were compared, both the first and second molars widths of archwires were found to be significantly narrower as compared to the first and second molar widths of the subjects. In females, the archwires showed significantly narrower CW, MW1 and MW2 width dimensions. The stiffness of an archwire is inversely proportional to the square root of its length. The more distant a tooth is from the midline, the lesser is the effect of the lateral expansion forces on the wire. For example, if the canine is approximately 12 mm and the first molar situated at 35 mm from the midline, the molar would receive only 13% of the lateral expansion forces as compared to the canine. Hence, the heavier NiTi archwires are more capable of changing the intercanine width during alignment and should be used with caution.

Other studies have reported both wider and narrower dimensions of the preformed archwires as compared to the arch form in our population.^21^ ^,^ ^22^ The differences in results may be due to ethnic variations and the choice of different archwire brands and shapes used in the various studies. In the present study, only archwires commonly used and easily available at various orthodontic centers across the country were evaluated. Moreover, as the arch form is defined by all the teeth, including canines, premolars and molars, in the present study the arch widths across premolars and second molar widths are also reported (Table 3). 

Over the years, various forms of archwires have been proposed based on linear parameters and various mathematical equations.^6^ ^,^ ^23^ ^-^ ^26^ The shape of the Bonewill-Hawley arch form is based on equilateral triangle, whereas the caternary arch form resembles a loop of a chain.^23^ These archwires have wider arch width dimensions at the second molar region. The Brader arch form, designed according to the forces of the surrounding musculature and narrower in the second molar region, was proposed some years later.^24^ The use of these archwire shapes may result in minimal undue modification and decreased crossbite tendency in the posterior region. As the shape of an archwire may affect its dimensions, these archwires were further classified into different categories on the basis of shape (Table 1). They were then compared individually with the mean arch dimensions of the mandibular arch in both males and females separately (Figs 3 and 4). In the current study, the Brader archwire shape in both males and females most closely conformed to the mean canine width of our population. The narrower shape of archwires, i.e. Bonewill-Hawley and tapered forms, most closely conformed to the first and second premolar widths and first molar widths in both males and females. In the second molar region, caternary and tapered arch forms in males and square arch forms in females most closely conformed to our subjects.

Hence, no single archwire shape from a particular manufacturing company conformed to the mean arch dimensions of our subjects. The use of the currently available preformed archwires may result in altered arch forms, increasing the tendency for post-treatment relapse. Ideally, NiTi archwires conforming to our population’s dimension should be manufactured and made easily available. Until these customized archwires for our population subjects become available, the dimensions of the most closely conformed archwires should be modified before utilizing them for our orthodontic practice. This may minimize the changes in the arch form of an individual, reducing the tendency for post-treatment relapse. 

## CONCLUSIONS

» Male subjects showed a trend towards increased arch depth and width, as compared to the female subjects.
» No single archwire conformed to the mandibular arch dimensions of the subjects included in the study.
» The mean archwire dimensions were generally found to be slightly wider at canine, first and second premolars widths.
» The mean archwire dimensions were generally found to be slightly narrower at first and second molar widths.
» Use of archwires that are too wide at canine level should be avoided. Arch width can be more easily controlled with formable archwires such as beta titanium (TMA) or stainless steel (SS).
